# A qualitative study on the clinical safety and user experiences of female condoms for anal intercourse among men who have sex with men in Shanghai, China

**DOI:** 10.3389/fpubh.2023.1243891

**Published:** 2023-11-23

**Authors:** Jia-Lu Huang, Xin Xin, Ming-Jun Ma, Zhen Ning, Shao-tan Xiao, Pan-pan Chen

**Affiliations:** ^1^School of Public Health, Dali University, Dali, China; ^2^Department of Epidemiology, School of Public Health, Fudan University, Shanghai, China; ^3^Pudong New Area Center for Disease Control and Prevention, Shanghai, China; ^4^Pudong Institute of Preventive Medicine, Fudan University, Shanghai, China; ^5^Shanghai Municipal Center for Disease Control and Prevention, Shanghai, China

**Keywords:** men who have sex with men, female condom, qualitative research, clinical safety, usage experiences

## Abstract

**Objective:**

This qualitative study aimed to understand the clinical safety, efficacy, and receptiveness of using the female condom (FC) during anal intercourse among men who have sex with men (MSM).

**Methods:**

Subjects for this study were recruited from a two-group crossover trial among MSM in Shanghai. The trial consisted of two phases, each including the use of condoms (FC vs. male condom), questionnaires, and in-depth one-on-one interviews. The two phases were separated by a washout period of 4 weeks. The minimum sample size for this study was determined in accordance with the principle of “information saturation.” The qualitative data were organized and analyzed using ATLAS.ti version 7.

**Results:**

A total of 26 participants from the MSM population were recruited for this study, with 10 assuming the insertive role (i.e., “1”), 8 assuming the receptive role (i.e., “0”), and 8 being versatile (i.e., “0.5”). Each participant completed the crossover trial comprising two phases. The cumulative usage of FCs and male condoms (MCs) amounted to 115 and 127 times, respectively. During the reported sexual encounters, no participants reported incidents of condom rupture, slippage, or other malfunctions. A few participants reported experiencing slight chafing pain, primarily put forward by “0” participants. Apart from those reports, no instances of bleeding, swelling, or allergic reactions were reported. The efficiency of FC in disease prevention, the sexual partner’s willingness to use FC, the freshness of FC, and positive sexual experiences were the main reasons for the consistent use of FC for anal sex. Discomfort and pain during sexual activity, the loose design and thick material of FCs, and difficulties in placing FCs were the major obstacles to FC use among MSM. The elements referring to the forehead exhibited varied in importance among “1,”, “0,” and “0.5” participants. Regarding the willingness to use the FC in the future anal intercourse, 61.54% of participants expressed a positive inclination, 23.08% were uncertain, and 15.38% stated that they would not. “A better sense of security during anal sex” was the main factor affecting willingness among “0” participants and “the sexual pleasure that the FC brought” among “1” participants. Improving the design and technology of FCs and increasing the frequency of use and practice might improve the use skills, which will favor the willingness to use FCs among the MSM population.

**Conclusion:**

FCs received positive user feedback from study participants, but distinctions were found in individuals in different sexual roles. Large-scale quantitative studies are needed to evaluate the clinical safety of the FC and its effectiveness in preventing the transmission of STDs during anal intercourse.

## 1 Introduction

HIV infection remains a serious global epidemic that impacts the vast majority of countries and regions (1). In China, the proportion of new HIV diagnoses transmitted through anal sex has been increasing for several decades, and the prevalence of HIV infection among men who have sex with men (MSM) remains high (2, 3). New HIV infections through homosexual transmission accounted for 0.9% of the cases in 2000, showing a dramatic increase to 25.5% in 2020 in China (4, 5). HIV prevalence in MSMs has grown to be a major public health challenge. Although the use of pre-exposure prophylaxis (PrEP) and postexposure prophylaxis (PEP) drugs may reduce the rate of HIV infection among the MSM population, it offers no defence against other STIs, such as syphilis, genital chlamydia, and gonorrhea (6). Additionally, studies have demonstrated failures and adverse drug reactions associated with PrEP and PEP (7), including nausea, vomiting, and renal failure (8). Furthermore, the widespread adoption of PrEP has been hindered by its exorbitant cost and limited availability (9). Compared with other preventive measures, condoms remain one of the most effective and cost-effective options. Currently, male condoms (MCs) are the most popular male-controlled contraceptive method, but they may impact sexual behaviors and sensations, notably on the sense of constriction and barriers, which lowers men’s level of sexual arousal and stimulation and affects their willingness to use condoms (10). The consistent and correct use of condoms remains low (11, 12), and high rates of unsafe sexual behaviors are observed among MSM (13). However, consistent and correct condom use is the most effective strategy for reducing sexually transmitted infections and improving protection rates among the MSM population (14–16). The female condoms (FCs), which is a barrier method contraceptive that is normally inserted into the vagina, are designed to address the issues associated with traditional male condoms (MCs). These products, similar to MCs, can be used by heterosexuals for both contraception and STD protection (13, 14, 17). FC is made of materials that are more durable than the typical MC; due to its loose design, it does not have the same level of constriction (18). As polyurethane materials have superior physical thermal conductivity to latex, using FC may have fewer negative effects on sexual arousal (19). In 2009, the FC2 female condom® (FC2) was approved by the U.S. Food and Drug Administration and is currently a commonly used and widely available FC. Meanwhile, studies investigating the acceptability and clinical effectiveness of FC have been conducted in various regions and populations worldwide, and the free promotion of FCs in many sub-Saharan African countries has increased the acceptance of the FC (20, 21). The research studies conducted in developed countries regarding the willingness to use FC during anal intercourse suggested a level of acceptability among the MSM population. Elizabeth Kelvin et al. who led an online investigation among 3,837 MSM living in the United States recently revealed a usage rate of 5.2% for FC during anal intercourse and identified several potential factors that might significantly influence the use of FC, including having multiple partners, being HIV-positive, and being on PrEP (22). However, there is still a need for more systematic and comprehensive studies to assess the clinical safety among MSM populations with different behavioral cultures, especially in developing counties (23, 24). To our knowledge on the literature currently available, in China, studies related to FC are limited and primarily conducted among couples or female sex workers, and there is no evidence of FC research in the MSM population (19, 25). The purpose of this in-depth interview was to gain insight into the potential use of FCs among MSM as well as to examine the clinical efficacy, safety, and willingness to use FCs among MSM. The findings aim to provide references for the development of targeted intervention strategies and measures.

## 2 Methods

This qualitative study described the results of in-depth interviews on the clinical safety and usage experiences of the FCs among MSM compared with the MCs.

### 2.1 Study design and population

**Figure 1:**
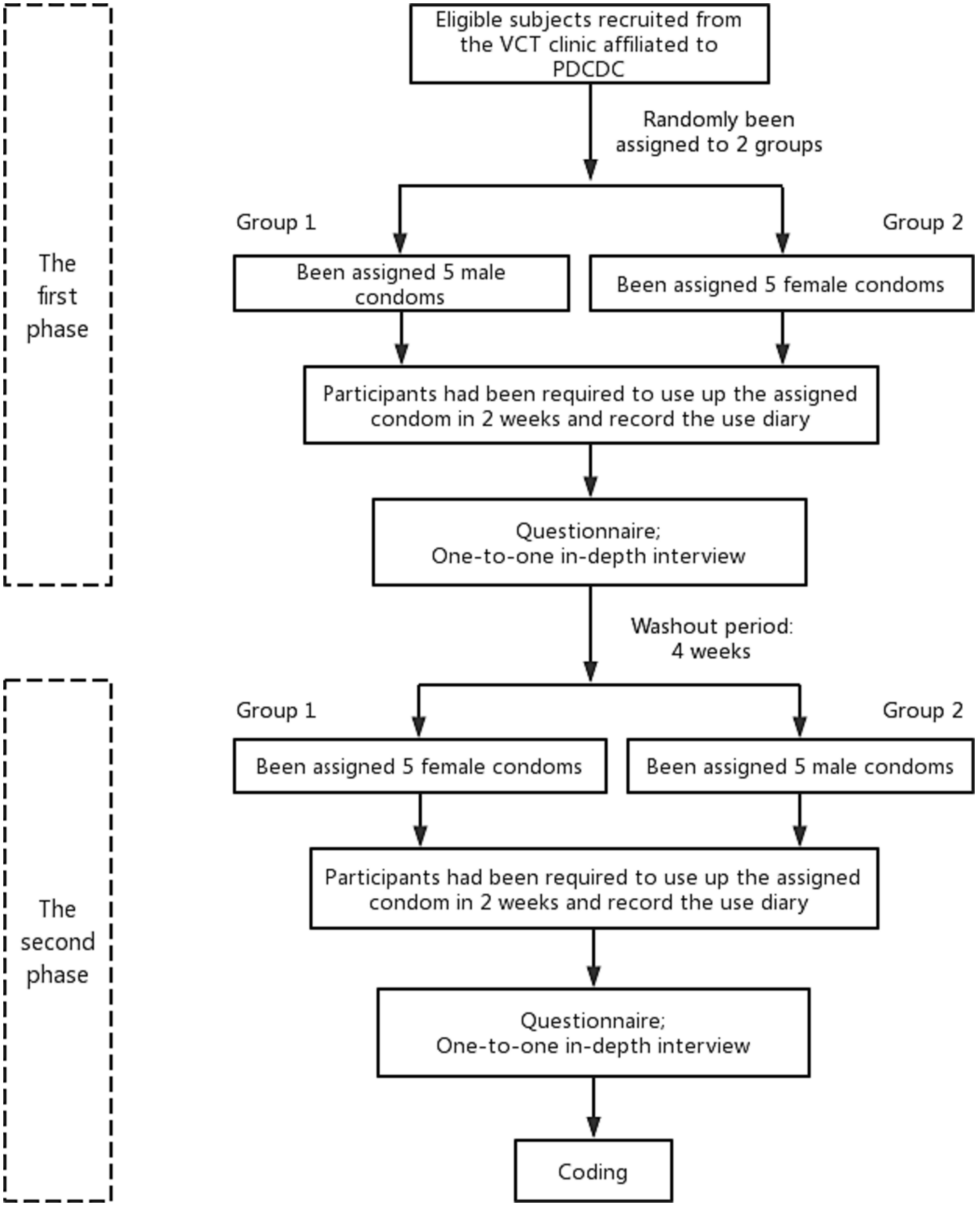
Flowchart of the two-phase crossover trial on the clinical safety and user experience of condoms among the MSM population.

Subjects of this study were recruited from a two-phase crossover trial on clinical safety and usage experience of condoms among MSM living in Pudong New Area, Shanghai, China. The participants were consecutively recruited from October to December 2021 at the HIV Voluntary Counseling and Testing (VCT) Clinic of the Pudong Center for Disease Control and Prevention (PDCDC). The inclusion criteria for the trial were as follows: male, aged ≥18 years, self-reported engagement in anal intercourse with at least one male partner in their lifetime, HIV negative, and provision of informed consent. Individuals with organic diseases or mental disorders were excluded. To obtain a more detailed and objective experience of FC use compared with MC, the participants were randomly divided into two groups for the two-phase cross-over trial. To eliminate the influence on the use experience and willingness to use an FC, based on the results of the preliminary experiment, a 4-week washout period was designed between the two phases. The 4-week period was a recommendation derived from the preliminary experimental results from the MSM population, prior to implementing the whole study. Moreover, it is operationally manageable within the context of the study and facilitates a convenient follow-up process. Each phase included the use of condoms (FC vs. MC), questionnaires, and in-depth one-on-one interviews. To ensure sufficient feedback on the experience and clinical data of condom use, the participants received training on the use of FC and MC before the condom trial. First, the essential usage process information relating to the two types of condoms was collected from the MSM volunteers in the preliminary experiment. Based on this, a comprehensive condom user manual was developed. Then, the user manuals were disseminated to all participants before they started to use the condoms. Subsequent to this, the participants received training concerning the correct method of using the FC as well as the MC, along with pertinent precautions. The trial included how to use FC during anal intercourse and how to handle condom-related issues, such as pain, bleeding, slippage, and other problems. Subsequently, the participants were assigned to different groups and received either five FCs or five MCs. Questionnaire surveys and qualitative interviews were conducted within 2 weeks after the participants reported using all of the assigned condoms. After a 4-week washout period, the second phase of the trial began immediately, in which the participants were instructed to switch condom types, with one group using MC in the first phase and FC in the second phase, while the other group followed the opposite sequence. The flowchart of the two-phase crossover trial is shown in Figure 1.

### 2.2 Data collection

The type of FC used in the crossover trial was the FC2 female condom®, while the MC used was the air series by Durex®. Participants were all demanded to keep a diary during the two-stage trials to help them record their respective experiences with each condom use and facilitated participants recall the details of their condom use more easily during subsequent in-depth interviews. In depth, one-on-one, face-to-face qualitative interviews were conducted in a separate room within 2 weeks after each phase of condom use. The duration of each interview ranged from 40 to 60 min. The minimum sample size for this study was determined based on the “information saturation” principle, which involved conducting interviews with 26 MSM participants until no new information could be obtained. Participants were recorded as “*DI01, DI02, DI03…*” according to the interview order for blurring the individual information. The interviews primarily focused on the clinical safety and usage experiences of condoms during anal intercourse. The usage experience covered aspects such as the steps of use, willingness to use, and barriers to usage. The contents of clinical safety included failure events and adverse reactions of FCs. Failure events encompassed rupture, slippage, misplacement, and inner ring displacement. Adverse reactions included bleeding, redness, and allergies. The topic questions and procedures of the qualitative interviews are presented in Table 1. Subsequently, the data collected from the in-depth interviews were gathered and organized. Prior to the start of the study, all participants read and signed informed consent forms, and all experiments were performed in accordance with the relevant guidelines and regulations of the Declaration of Helsinki.

**Table 1:** Interview topic guides for MSM.

Topic	Example questions or description
Introduction interview	Interviewer introduced the interviews
Informed consent	Verbal and written consent was provided
Clinical safety of using the FC	
Failure events	“How would you describe the failure events when using the FC?”[rupture; slippage; misinsertion of penis; outer ring been pushed into the anal canal; misplacement] *Prompt how did the failure events influenced sexual behaviors.* How did [insert aspect mentioned] influence sexual behaviors, and what were the outcomes of [insert aspect mentioned]?
Adverse reactions	“How would you describe the adverse reactions when using the FC?”[bleeding; allergy; ache] *Prompt how the failure events influenced sexual behaviors.* How did [insert aspect mentioned] influence sexual behaviors, and what were the outcomes of [insert aspect mentioned]?
The experience of using the FC compared with MC	
Usage experiences	“What’s your opinion on the impact of the HIV infection and other STDs on your life?”
	“What is the role of condoms in your sexual behaviors?”
	“What is your opinion on the use experience and procedure of MC and FC?”
	“What are the advantages of the MC and FC?”
	“What are the disadvantages of the MC and FC?”
	“What is your perception on the use effects of the MC and FC?”
Willingness of FC using	“What is your willingness to use the female condom during ANAL SEXUAL BEHAVIOR in the future?”[willing to use; definitely not use again; uncertain] What are the causes [insert aspect mentioned] influencing the willingness to use the FC?
Closing	Closing of the interview

### 2.3 Data analysis

All qualitative data were transcribed verbatim in Chinese, and each interview transcript was reviewed by two team members (i.e., double coding). The data were organized using ATLAS.ti version 7, a qualitative data management software. To determine the clinical safety and usage experiences of the condoms used by MSM participants, content analysis was conducted using ATLAS.ti to categorize and analyze the interview reports of the 26 MSM participants. Content analysis provided a theoretical framework for understanding the actual content. It was used to make objective inferences about subjects of interest or to identify certain words, phrases, features, or sets of texts in an objective manner (26). Using open coding, all texts related to the use of condoms (FC vs. MC) were coded into subcategories by three investigators. These subcategories were further organized into families to form categories. For quality control and to ensure the interpretive proximity to the content (27), the researchers repeatedly compared transcripts between different categories and subcategories of “usage experiences, clinical safety, and other sorts.” Regular discussions among coders further supported the reflexivity of the analysis process and its outcomes.

### 2.4 Sensitivity analyses

To evaluate the robustness of our findings, sensitivity analysis was conducted by selecting participants who completed the entire two-phase crossover trial and participated in both groups.

## 3 Results

A total of 26 MSM participated in this qualitative research and completed in-depth interviews. A total of 115 FCs and 127 MCs were used. Among the study participants, 11 were between the ages of 20 and 29, 7 were between 30 and 39, 5 were between 40 and 49, and 3 were 50 years old or older. In terms of occupation, three participants were in catering management, service industry, sales promotion, IT industry, construction industry, and student. Two participants were employed in the medical field, enterprise, trade business, and public institutions. Based on the position for anal sexual (i.e., top, bottom, versatile), 10 participants identified as tops (referred to as “1”), 8 participants identified as bottoms (referred to as “0”), and 8 participants identified as versatile (referred to as “0.5”). In terms of education, 5 participants had a junior high education or less, 7 participants had completed high school, 11 participants had a bachelor’s degree, and 3 participants had a postgraduate degree.

### 3.1 Clinical safety

During the interviews, we gained in-depth insights into the failure events and adverse reactions to FCs experienced by MSM during anal intercourse.

#### 3.1.1 Failure events

##### 3.1.1.1 Rupture

None of the participants reported experiencing FC rupture. Approximately two-thirds (17/26) of the interviewees considered the FC to be sturdy and of good quality and expressed no concerns regarding rupture or tearing.

“FC was very strong, stronger than MCs and had good quality. There was no breakage or cracking, and I'm sure there won't be this problem.” (DI02, IT industry, 26 y, “0.5”)

##### 3.1.1.2 Slippage

The duration of FC usage during anal intercourse reported by the interviewed MSM participants ranged from 15 to 40 min, and the majority of the participants did not perceive slippage as a significant issue. Twenty-one participants mentioned that the FC remained securely in place throughout sexual intercourse, with no instances of slippage. Some participants (9 of “0” vs. 2 of “1”) expressed concerns about slippage, stating that it could be distracting during sexual activity. Referring to MC, 3 of the “0” role participants mentioned their concern that the “1” sexual partners might pull out of the condom without notice during sex; they were less anxious when using the FC as the situation would be noticed immediately during the use of FC.

“I looked at it, and it didn't slide out.” “I had worried that the FC would fall out the whole time, and that had distracted my attention on sex.” (DI22, student, 25 y, “0.5”)

“If my friend (sexual partner) tries to remove the FC halfway, I could find it as owing to the feeling from inside.” (DI17, waiter, 22y, “0”)

##### 3.1.1.3 Incorrect insertion of the penis

A total of 16 subjects used the FC as the “inserter” during anal intercourse, with a total of 70 instances, and no cases of incorrect insertion were reported. Eight participants mentioned having difficulty with the initial insertion, stating that it was not very smooth. The remaining 8 participants felt that the insertion was not problematic because they employed certain techniques such as enemas or dilation.

“The insertion was smooth as some tricks were used. You have to expand the anus first. We all had done that. Of course you can't just put it (FC) in.” “Put some lube around it (FC) before you insert it.” (DI08, quality inspection, 25 y, “0.5”)

##### 3.1.1.4 The outer ring falls in

All participants reported no issues with the outer ring being pushed into the anal canal, and they did not express concerns about this occurrence.

“How is that possible? The outer ring was very big. It's impossible. It (outer ring) was very stable, and the area outside was quite large.” “There was no falling in.” (DI10, Construction industry, 45 y, “1”)

##### 3.1.1.5 Displacement

The majority of participants believed that they or their partners were able to position the FC correctly. Six participants (none of them were “1”) mentioned experiencing difficulties during the first or subsequent uses, but some of them stated that they became more proficient after watching the instructional video or practising. Participants found it easier to position the FC after using enemas, anal dilation, or lubricants.

“At first, I did not watch the teaching video beforehand, and I did not know how to use it (FC). Then, I watched the video and found that when using the FC I have to apply lubricant. Applying the lubricant is indeed much better.” (DI25, service industry, 33 y, “0”)

“After the enema, it will be much looser, and it will be easy to wear the FC. Enemas were common among MSM, even when using the MC.” (DI26, HR industry, 28, “0.5”).

“At the beginning, as the lube was not enough, I did not feel very easy to place FC, and then I used some lube.” (DI05, Service industry, age 33 y, “0”)

#### 3.1.2 Adverse reactions

##### 3.1.2.1 Bleeding

None of the subjects in this study reported experiencing bleeding.

“I did not find bleeding, allergy or achy feeling. It (FC) was soft, and it was unlikely to break the skin.” (DI05, Service industry, 33 y, “0”)

##### 3.1.2.2 Allergy

None of the subjects reported allergies.

##### 3.1.2.3 Pain

Seven of the “0” mentioned that they experienced pain when the FC was used in receptive sexual, behavior and that the pain lasted longer. The main reason for the pain was attributed to the inner ring. Three of “1” mentioned experiencing pain when using FC, but they considered the pain to be mild and tolerable, with minimal impact on sexual activity.

“It felt like the first time I had anal sex, bloated and a bit uncomfortable, it affected me a lot.” (DI11, Student, 22 y, “0”)

“I felt comfortable after putting it (FC) in, but my friend would still feel a little uncomfortable because for the inner ring.” (DI03, Food and Beverage Management, 23 y, “1”)

“I touched the ring inside occasionally and felt uncomfortable, but it was slight and I used the FC until the sexual intercourse finished.” (DI01, Sales, 28 y, “1”)

#### 3.1.3 Solutions to the failure events and adverse reactions

All participants reported that they had switched to MCs and continued their current sexual activities. None of the participants engaged in unprotected anal intercourse when encountering failure events or adverse reactions, and there were no instances of discontinuation of sexual activity.

### 3.2 Usage experiences

Among the 115 instances of FC use, full-course use occurred 81 times, which accounted for 70.43% (81/115) of the total. Among the 34 instances of partial usage, 61.77% (21/34) stopped using the FC after the first time, 29.41% (10/34) stopped after the second time, and 8.82% (3/34) stopped after the third time. There were no reported instances of failure in using MC. The following shows an overview of the usage experiences of the FCs compared with MCs in anal intercourse among MSM, including the reasons for both full and partial usage.

#### 3.2.1 Reasons for using FC throughout the entire duration

##### 3.2.1.1 The FC is efficient in disease prevention

Seven of “0,” 1 of “0.5,” and 2 of “1” mentioned that the texture of FCs appears to be safer; thus, they insisted on using them up. Several participants stated that FCs have a larger outer ring, which avoids direct contact with their partner’s skin.

“The effect is OK. I like the outer ring design and I feel FC is safer than the MC.” (DI05, service industry, 33 y, “0”)

“My friend asked me whether the last condom (FC) was still available, and he thought it was good. So, we used it again.” (DI14, Construction industry, 50 y, “1”)

##### 3.2.1.2 Freshness on FC

Seven of the “1” participants and 5 of the “0” participants stated that despite some discomfort with using FCs, they still persisted in using them because they were curious about them.

“Maybe he (sex partner) felt uncomfortable at first, but both of us were very curious.” (DI06, Trade, 39 y, “1”)

“I used it (FC) throughout the sexual process. I had never see it (FC) before, and it looks like a little funny. I was courious about the feeling during the use of FC” (DI25, service industry, 33 y, “0”) [sic]

##### 3.2.1.3 Positive sexual experience

Five of the “1” participants and 2 of the “0” participants indicated that the FC performs well and is a positive experience.

“FC is looser. It felt just like going without wearing a condom.” “Been done with FCs, did not change.” (DI07, Food and Beverage Management, 35 y, “1”)

#### 3.2.2 Reasons for not using the FC throughout sex

##### 3.2.2.1 Discomfort and pain during sexual activity

Eight of “0” participants and 3 of “1” participants cited discomfort and pain as the main reason for not using the FC throughout anal intercourse. The users claimed that when using the FC, they experienced discomfort mainly due to the sensation of a foreign object in the anal canal.

“FC was not comfortable for my friend and I felt uncomfortable a little painful and strange. I had been accustomed to using the MC.” (DI01, Sales, 28 y, “1”)

“I tried (FC), but i still didn’t feel good, especially the feeling of pain.” (DI11, Student, 22 y, “0”)

##### 3.2.2.2 Loose design and thick material of FC

Six of “1” participants, 1 of “0.5” participants, and 1 of “0” participants mentioned that FCs were thicker and affected sexual pleasure; that is, FCs are thick and anal intercourse is less enjoyable.

“My friend said the FC was a little thick, and there was no sexual pleasure, I myself also felt it was a little thick.” (DI07, Food and Beverage Management, 35 y, “1”)

##### 3.2.2.3 Difficulties in properly placing the FC

Five of “0” described difficulties in placing FC due to the large inner ring. One of “0.5” expressed uncertainty about whether the condom was placed correctly and whether the anus would push the FC out.

“The FC was not placed well twice. Anyway, after ten minutes of use, I still felt uncomfortable, so I took it (FC) out and replaced it with an MC.” (DI16, IT industry, 40 y, “0”)

“As the FC was stuck inside, I did not know whether it had been put in a good position, and there was a foreign body and uncomfortable feeling. So, I took it (FC) out directly, and we did not succeed in using this FC.” (DI20, Medical industry, 28, “0.5”)

##### 3.2.2.4 Sexual partner’s unwillingness to use the FC

Two of “1” and 3 of “0” mentioned that they did not use the FC throughout because their partner did not want to continue using the FC.

“I thought it (FC) was OK, but my friend felt uncomfortable. Then he took it off and asked me to wear the MC instead.” (DI23, Service industry, 52 y, “1”)

### 3.3 Willingness to use

A total of 30.77% of participants (8/26) expressed willingness to try the FC again in the future and to use it as a supplement to MC. A total of 15.38% of participants (4/26) explicitly stated that they would not be willing to try the FC again. A total of 23.08% of participants (6/26) did not clearly indicate whether they would use the FC again in the future and whether they would continue to use the FC is related to the type of sexual partner they would have.

#### 3.3.1 Reasons for the willingness to use

Six of “0,” 4 of “0.5,” and 3 of “1” participants mentioned that the large outer ring of the FC avoids direct contact with the skin and body fluids of the sexual partner, bringing a better sense of security during anal sex. Five of the “0” and 2 of the “0.5” participants thought they would use the FC, instead of MC during anal sex when their sexual partner refused to use the MC. The willingness to use the FC in the “1” role comes more from the sexual pleasure that the FC provides as it is less constricting and makes for a better sexual experience than MC.

“If this condom is still available in the future. I'll use it. It (FC) will protect me from getting infected with HIV. If my friend removies the FC halfway through sex. I can feel that.” (DI25, service industry, 33 y, “0”) [sic]

“I think it (FC) was pretty good, and I will definitely use it (FC) again…I could use the FC if my friend doesn’t want to use an MC.” (DI12, Medical industry, 31 y, “0”)

“FC is comfortable, and I like it. So did my friend.” (DI07, Food and Beverage Management, 35 y, “1”)

#### 3.3.2 Reasons for the unwillingness to use

Two of the “0” participants complained of the complexities of the use of FC, such as the difficulty of placement, and these obstacles influenced their willingness to use FC. Swelling and pain were associated with FC during sexual activity. Four of the “1” participants stated that they were more accustomed to using MC due to the shortcomings of FC design, such as the loose body of FC. The subjects also stated some situations for improving their willingness to use FC. For example, improving the design and technology of FC, especially the softness of the inner ring, would reduce the occurrence of pain. Increasing the frequency of use and practice might improve the use skills, thus adding confidence in the successful use of FC.

“…but there were too many steps during using the FC…and maybe I was too sensitive to the feeling of pain.” (DI11, Student, 22 y, “0”)

“…the feeling of FC was not the kind of desired feeling. I may not use it again, unless the outer ring were, softer and the body of the FC tighter.” (DI10, Construction industry, 45 y, “1”)

“I don’t plan to use the FC again, but I think more practice on placement would improve the possibility of successful use.” (DI03, Food and beverage management, 23 y, “1”)

## 4 Discussion

The risk associated with MSM engaging in unprotected anal sexual activity is an increased vulnerability to infection with HIV and other STDs (27–29). In light of this, using condoms is still the simplest and most efficient preventative strategy. The percentage of men consistently using MC during anal intercourse remains poor due to male reluctance, allergies, and other factors. FCs were initially designed as an alternative to MCs, with the majority of investigations on the acceptability and safety of FCs undertaken by women. Few studies have focused on anal sex among MSM (30). It is difficult to ascertain conclusions regarding the safety of using FCs given the limited available literature regarding their usage for anal sex (31, 32). This study collected in-depth interview information through qualitative research on the clinical safety and usage experiences of FCs during anal intercourse among the MSM population. Each participant in this study had successfully completed a crossover trial using the FC and MC to have our results regarding FC contextualized by the widespread use of MC.

Our study did not find failure events such as “rupture,” “slippage,” “outer ring falls in,” and “minsertion.” The lack of failure events in our study may be attributed to the FC’s instruction manuals and videos prepared in advance and MSM were asked to refer to these supporting materials prior to using the FC. By properly training the individuals, we were able to ensure proper use, which contributed to the lowered likelihood of failure (33). Although the FCs were initially designed for vaginal insertion, the negative reactions to the FC in anal sex were relatively low, and there were no adverse reactions, such as allergic reaction and bleeding, reported in this specific study. The swelling pain likely caused by contact with the inner ring was the main negative reaction found in our study (34), implying that the improvement of the inner ring design of an FC is needed (22). Although FCs can be worn during anal intercourse with or without the inner ring, removal of the inner right will impact retention leading to a slippage (32, 35). Meanwhile, previous studies conducted among female subjects have indicated that slippage may be the most prevalent type of clinical failure (36, 37). Some studies have documented that the possibility of failure events decreases with more frequent use of the FC (38, 39). Combined with the previous findings and the results of our study, these findings indicate that additional quantitative studies are needed to explore the frequency of failure events and negative reactions to FCs used among MSM, and the factors influencing failure events and adverse reactions to achieve a wider use of FCs (40). However, several participants reported that FCs required more care than MCs during intercourse, which affected their sexual performance due to the fear of failure events (i.e., slippage), and adverse reactions (i.e., pain) (41). Notably, the “0” participants might express more concerns during the use of the FC. Thus, more attention should be given to the concerns of the occurrence of failure events, especially the crowd of “0” participants. In our study, respondents who did not use FCs reported switching to MCs, showing that the strategy of adding the use of FCs (vs. MCs only) might result in a higher rate of overall condom use among the MSM population.

Insights can be drawn from the experience of promoting the FC, where acceptability and usage experience have a significant impact on compliance (10). This study has shown that the majority of participants used FCs throughout sex with positive experiences mainly stemming from the idea of safer sex, enjoyable sexual experiences, and overall curiosity about the FC (42, 43). However, after comparing MSM in various roles, there are disparities in experience and willingness to use FCs. The primary motivation for the “0” role to use FC is self-defence, to prevent contracting STDs and AIDS. In contrast, the “1” participants care more about the excitement and pleasure of the sexual experience provided by FCs. For example, the loose design of the FC can alleviate a sense of restriction to some extent (44). In the past, MSM had access only to MCs, but this study has proven that FCs might be a relatively effective and safe alternative barrier tool to prevent the spread of sexually transmitted diseases. Therefore, the FC appears to be a complementary option to male condoms. Accordingly, the consistent use of FCs during anal sex arises as a non-negligible issue. Our study found that partner willingness plays a significant role in the overall use of FCs among MSM, which is consistent with findings from other studies (37). In particular, according to the view of “1” participants, the use willingness of “0” participants is an important reason to enhance their overall use of FC, while the efficiency of FC comes first among the “0” participants (45). The decision on the use of MC is often held by the “1” participants, which is a vital limitation of the current condom promotion strategy among MSM. Considering that the role “0” would be more susceptible to infection from unsafe sexual activities, the FC could provide the “0” participants the initiative to better protect themselves (40, 46). The primary obstacles pointed out by the study on utilizing FCs in the MSM population are placement and insertion problems (47). In our study, the biggest barriers to using FCs among “0” participants were the inconvenience of placement and the interference with sexual enjoyment. Optimizing the FC design and providing instructions tailored to the usage issues observed in the MSM population may improve the user experience.

### 4.1 Strengths and limitations

This study is the first to indicate the clinical safety of the use of FCs by MSM, discuss the willingness of FC to use FCs among MSM living in China, and provide a reliable basis for the use of FCs in anal sex among MSM. As MCs are widely available and FCs are more expensive than traditional condoms, the study did not mention the effect of access to FCs on acceptance (48). Then, qualitative studies could not evaluate the frequency of occurrence of each event for clinical safety, which needs further quantitative studies with larger sample sizes.

The influence of the appearance of FCs on acceptability was not broached in the interviews with study participants. According to certain research, the outer ring of an FC outside the anal canal would have an esthetic impact, which was a major factor in its rejection (49).

## 5 Conclusion

In conclusion, MSM is referred to as a bridge population and are vulnerable to various kinds of sexually transmitted diseases, including HIV, due to their complex sexual relationships and high-risk sexual behavior (29). Controlling HIV infection among MSM requires a combination of various prevention interventions to acquire better results. Our research suggests that MSM people have a clear willingness and demand for FCs, which is considered as a complementary and alternative to MCs. Differences in usage experience and willingness existed among various sexual roles. “0” participants’ adverse experiences with using FCs were primarily due to the pain caused by the inner ring; however, the need for disease prevention increased “0” participants’ willingness to use FC. While “1” participants place more emphasis on the freshness and pleasurable sexual experience provided by FCs. FCs might be an important public health initiative to increase the proportion of safe sexual behaviors, but extra attention should be given to the sexual roles during the promotion or application of FCs among MSM. A further quantitative study aiming at clarifying the clinical effectiveness and safety of FCs during anal sex is needed.

## Data availability statement

The original contributions presented in the study are included in the article/supplementary materials, further inquiries can be directed to the corresponding author.

## Ethics statement

The studies involving human participants were reviewed and approved by the Ethics Committee of Pudong New Area Center for Disease Control and Prevention. Written informed consent was obtained from the individual(s) for the publication of any potentially identifiable images or data included in this article.

## Author contributions

J-LH and XX conceived the study and contributed equally to this study. J-LH was responsible for preparation of the figures and manuscript writing. P-pC recruited participants. P-pC and XX interpreted data and reviewed articles. J-LH, XX, and P-pC polished the manuscript language. XX, M-JM, and ZN aided in the recruitment of participants. P-pC conceived the design of the experiment and organized the whole progress of the project. S-tX provided financial support for this study. All authors contributed to the discussion of the data and to the manuscript.
